# The relationship between work–family conflict and job burnout among primary and secondary school teachers: the role of depression and cognitive reappraisal

**DOI:** 10.3389/fpsyg.2024.1438933

**Published:** 2024-09-24

**Authors:** Yue Li, Xingcan Ni, Wei Zhang, Jianping Wang, Chengfu Yu, Hongyu Zou

**Affiliations:** ^1^College of Teacher Education, South China Normal University, Guangzhou, China; ^2^Department of Psychology, Research Center of Adolescent Psychology and Behavior, School of Education, Guangzhou University, Guangzhou, China; ^3^Center for Studies of Psychological Application, School of Psychology, South China Normal University, Guangzhou, China; ^4^Journal of South China Normal University, South China Normal University, Guangzhou, China

**Keywords:** work–family conflict, job burnout, depression, cognitive reappraisal, school teachers

## Abstract

**Background:**

Primary and secondary school teachers are a high-risk group for job burnout, and how to alleviate their job burnout has become an increasingly urgent issue. Previous studies have paid less attention to the differential effects of the bidirectional interaction between work and family on the job burnout of the teachers. This study aim to explore the different impact of work–family conflict and family–work conflict on job burnout among primary and secondary school teachers, as well as its underlying mechanisms.

**Methods:**

This study selected 2,184 primary and secondary school teachers in China (Mage = 37.26; SD = 9.40) as participants using a random sampling method. Using the SPSS Process 4.0 macro plugin constructed a moderated mediation model, the study explored the relationships between two different forms of work–family conflict, depression, cognitive reappraisal, and job burnout.

**Results:**

The study results indicated that both forms of work–family conflict were significantly positively related to the job burnout, and this relationship was influenced by the mediating role of depression. Furthermore, cognitive reappraisal moderated the relationship between depression and job burnout.

**Conclusion:**

This study revealed the potential pathways influencing job burnout among primary and secondary school teachers in the Chinese cultural context. Focusing on and alleviating work–family conflicts for primary and secondary school teachers is crucial for mitigating their occupational burnout. Additionally, teachers should also carefully and reasonably use cognitive reappraisal as an emotional regulation strategy to adjust the impact of depression on occupational burnout.

## 1 Introduction

In recent years, job burnout has increasingly become a major concern for many people, and teachers’ job burnout may jeopardize various aspects of themselves and their students (Maslach and Leiter, 2008; Madigan and Kim, 2021b). Job burnout refers to a state of depletion in individuals caused by excessive stress related to work in physiological, mental, emotional, and behavioral aspects (Fuming, 2003). It is a reaction to prolonged stress related to work, specifically characterized by emotional exhaustion, depersonalization, and diminished personal accomplishment (Maslach and Leiter, 2016). Job burnout has become a significant issue in the teaching profession. In a recent study, 43.26% of teachers self-reported experienced job burnout (Xue et al., 2023). Compared to other professions, primary and secondary school teachers are more prone to job burnout due to excessive workload and/or conflicted demands, as well as faced multiple pressures from parents, schools, and society (Iancu et al., 2018; Rasanen et al., 2022). Job burnout has a wide-ranging impact on teachers, damaging their interpersonal relationships, quality of life, teaching quality, job satisfaction, intention to leave, and physical and mental health. Furthermore (Capone et al., 2019; Garcia-Carmona et al., 2019; Madigan et al., 2023; Madigan and Kim, 2021a), it also affected students’ academic performance, learning motivation, mental health, and social and behavioral issues (Madigan and Kim, 2021b). Therefore, it is necessary to delve deeper into the issue of Job burnout among primary and secondary school teachers.

When exploring the causes of teacher Job burnout, we find that various factors such as work environment, professional skills, psychological capital, and sense of efficacy played important roles (Candeias et al., 2021; Capone et al., 2019; Pu et al., 2017). Among these, the relationship between teacher’s work and family, especially work–family conflict, was a very significant aspect that is increasingly receiving attention from researchers. Work–family conflict was a form of role conflict triggered by role pressures at the work and family levels in terms of resources, emotional expressions, and behavior (Greenhaus and Beutell, 1985). It included work-to-family conflict (WFC) caused by job demands and family-to-work conflict (FWC) caused by family demands (Frone et al., 1997a). According to the conservation of resources theory (Grandey and Cropanzano, 1999), individuals had limited resources in both work and family domains. When individuals expended too many resources in one domain that they are unable to invest enough resources in the other, work–family conflict arises. Prolonged conflict led to a sense of resource depletion, prompted individuals to reduce resource investment at work to conserve existing resources, thus led to job burnout (Hobfoll, 2002). Numerous empirical studies also highlighted the significant impact of work–family conflict on teacher job burnout (Junca-Silva and Freire, 2022; Pu et al., 2017; Shang, 2022). Family and work were indispensable parts of primary and secondary school teachers’ lives, and a dynamic balance should be achieved (Brosch and Binnewies, 2018). When they struggled to cope with the dual roles of work and family, work–family conflict became a major challenge, not only created additional stress but also exacerbating the tendency toward job burnout (Simaes et al., 2021; Lambert et al., 2022).

However, most of the past research on work–family conflicts has overlooked the interactions between work and family, focused more on single dimensions or overall conflict. There was still a lack of understanding of the differential effects of WFC and FWC and their underlying mechanisms (Li et al., 2021). In fact, work and family are mutually influential, so WFC and FWC may have different impacts on primary and secondary school teachers. We should fully understood the work–family conflict from a bidirectional perspective (Tone Innstrand et al., 2008), explored the different effects of the two types of work–family conflicts on job burnout and their potential mechanisms to provide targeted recommendations for alleviating job burnout among primary and secondary school teachers and improving educational quality. Based on the above considerations, we focused on primary and secondary school teachers as our research subjects, studying the impact of the two dimensions of work–family conflict – WFC and FWC on their job burnout. We hypothesized that different forms of work–family conflict are significantly positively correlated with job burnout among primary and secondary school teachers, and there may be differences in their potential mechanisms.

It is worth noting that, even under the same working conditions and family demands, not all individuals will experience the same level of job burnout (Simaes et al., 2021). Therefore, understood the individual factors influencing job burnout and the role these factors may play in the interplay between work–family conflict and job burnout is crucial (Pu et al., 2017). Depression symptoms, as a typical negative emotional state, were prevalent in various aspects of life, manifested by symptoms such as sleep disturbances, fatigue, lack of concentration, loss of appetite, and lack of interest in daily experiences (American Psychiatric Association, 2013), led to impaired interpersonal relationships, social, and occupational functioning (Ibrahim et al., 2013). Despite the close association between job burnout and depression symptoms, some past studies have regarded them as overlapping concepts (Bianchi et al., 2021); however, a recent network analysis indicated they have distinct structures (Wang et al., 2024). According to the affective events theory (Weiss and Cropanzano, 1996), experienced negative work events triggers individual emotional responses, further influencing attitudes and behaviors. Therefore, when individuals face frequent work–family conflicts (a form of negative work event), they may experienced intense negative emotions (Zhou et al., 2018). If these emotions persisted and gradually infiltrated into work, individuals may experienced the same negative emotions in their work, reduced work enthusiasm and motivation, ultimately led to job burnout. Consistent with this viewpoint, empirical evidence indicated a positive correlation between work–family conflict and depression, with individuals experienced severe work–family conflict showed high levels of depression (Peter et al., 2016; Wang and Peng, 2017; Zeng and Guo, 2024; Zhang et al., 2023). Furthermore, depression can lead to a reduction in the psychological resources available to individuals at work, made it difficult to cope with job demands, thereby increased job burnout (Hatch et al., 2019; Zeng and Guo, 2024). Based on the theory and empirical evidence, this study assumed that depression can significantly mediate the relationship between two types of work–family conflict and job burnout among primary and secondary school teachers.

The stress-buffering model (Cohen and Wills, 1985) suggested that protective factors can weaken the impact of risk factors. According to this theory, not all individuals affected by work–family conflict will experienced negative emotional states, such as job burnout (Simaes et al., 2021). Teachers in primary and secondary schools can use cognitive resources to help them cope with these challenges (Weiss and Cropanzano, 1996). Cognitive reappraisal was an adaptable emotional regulation strategy where individuals change their understanding of emotional events and the personal significance of these events to regulate emotions (McRae et al., 2012). Previous studies have highlighted the numerous benefits of cognitive reappraisal in the workplace; it can reduce negative emotions, increase job satisfaction, and prevent burnout (Feinberg et al., 2020; Shapero et al., 2019). Therefore, teachers who use cognitive reappraisal to minimize or avoid negative emotional experiences may be less affected by these emotions, thus reducing burnout. Given that cognitive reappraisal was primarily flexible and effective in reducing negative emotions such as depression and led to more positive emotional outcomes (Gross, 2014), this study focused on the impact of cognitive reappraisal on depression, assuming that cognitive reappraisal can significantly moderated the relationship between depression and job burnout.

**Figure 1:**
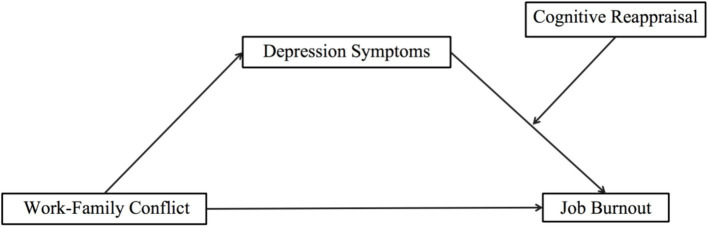
Hypothesis model.

In summary, this study will, for the first time in a Chinese context, explore the relationship between two different forms of work–family conflict and job burnout among primary and secondary school teachers based on the affective events theory and the stress-buffering model. It will also examine the roles of depression and cognitive reappraisal in this relationship. The findings of the study will have significant theoretical and practical implications for understanding and uncovering the mechanisms behind occupational burnout among primary and secondary school teachers (Figure 1).

Based on the literature review, this study proposes the following hypothesis:

*H1*: Both forms of work–family conflict are significantly positively correlated with job burnout among primary and secondary school teachers.

*H2*: Depression can significantly mediated the relationship between two different forms of work–family conflict and job burnout.

*H3*: Cognitive reappraisal can significantly moderated the relationship between depression and job burnout.

## 2 Methods

### 2.1 Participants and procedure

This study utilized a random sampling method to gather data through 2,514 online questionnaires distributed in April 2023 across Guangdong Province in South China, using the Wenjuanxing platform.^1^ All participants were primary and secondary school teachers who could fill out the questionnaire by scanning a QR code or clicking on a link. After excluding incomplete responses, surveys completed in less than 210 s, and questionnaires with abnormal ages, a total of 2,184 valid questionnaires were collected (505 males, 23.12%). The effective response rate was 86.87%. The average age of the participants was 37.26 years with a standard deviation of 9.40, ranging from 18 to 61 years.

Among the valid respondents, 2,056 held undergraduate degrees, 210 possessed postgraduate degrees, and 248 had associate degrees. Notably, 1,805 teachers (71.80%) were employed in public schools. Participation in this study was entirely voluntary, allowing participants the freedom to opt in or out as they chose. Before taking part, all individuals signed a written informed consent form online. This study was approved by the Ethics Committee of the School of Education, Guangzhou University.

### 2.2 Measurements

#### 2.2.1 Work-to-family conflict and family-to-work conflict

Work–family conflict and family–work conflict were assessed using the work–family conflict questionnaire developed by Carlson et al. (2000). The questionnaire was adapted into Chinese by Lu et al. (2009) and consists of 12 items such as “I have to sacrifice time with my family to complete necessary work tasks.” Responses were rated on a Likert scale from 1 = strongly disagree to 5 = strongly agree across two dimensions—work–family conflict and family–work conflict. A higher total score indicated a higher level of conflict. In this study, the questionnaire demonstrated good reliability with a Cronbach’s *α* coefficient of 0.89.

#### 2.2.2 Depression symptoms

Depressive symptoms were assessed using the PHQ-9 scale developed by Kroenke et al. (2001) and introduced into Chinese by Wang et al. (2014). This scale measures the presence and severity of depressive symptoms with 9 items (e.g., feeling down, depressed, or hopeless). Responses were scored on a 4-point Likert scale ranging from 0 (not at all) to 3 (nearly every day), with a total score ranging from 0 to 27. A higher score indicated more severe depressive symptoms. The questionnaire demonstrated good internal consistency with a Cronbach’s *α* coefficient of 0.92.

#### 2.2.3 Job burnout

Job burnout among primary and secondary school teachers was assessed using the Job Burnout Questionnaire developed by Fuming (2003). The Likert-type scale ranged from 1 = strongly disagree to 5 = strongly agree, comprising 15 items such as “I feel overburdened at work.” The total score indicated the level of occupational burnout among teachers, with higher scores reflecting higher levels. In this study, the Cronbach’s α coefficient was 0.71.

#### 2.2.4 Cognitive reappraisal

Cognitive Reappraisal was measured using the Cognitive Reappraisal dimension questionnaire from the Emotion Regulation Questionnaire developed by Gullone and Taffe (2012) and translated into Chinese by Liu et al. (2017). The scale consisted of 10 items, with the Cognitive Reappraisal dimension comprising 6 items (e.g., “When I want to feel happier, I think about something different”), specifically items 1, 3, 5, 7, 8, and 10. Responses were rated on a Likert scale from 1 for “completely disagree” to 5 for “completely agree,” with higher scores indicating greater use of cognitive reappraisal strategies. The questionnaire demonstrated a high internal consistency with a Cronbach’s α coefficient of 0.94 in this study.

### 2.3 Statistical analysis

Data organization and preliminary analysis were performed using IBM SPSS 26.0. Common method bias was assessed through Harman’s single-factor test. Factor analysis was used to test the covariance for data. Then, the Shapiro–Wilk test was used to test the normality of each continuous key variable. Variables were subjected to, descriptive statistics, with normal data described by mean plus or minus standard deviation. Spearman correlation analysis was also performed to explore the correlation between the main variables.

The Moderated mediation model was established using the Process 4.0 macro plugin in SPSS. Hypotheses 1 and 2 were tested using the mediating model (Model 4), while hypothesis 3 was tested using the moderated mediation model (Model 14). Both models controlled for gender and age effects. When examining the model of work–family conflict on job burnout, family–work conflict was controlled; and when examining the model of family–work conflict on job burnout, work–family conflict was controlled. The independent, dependent, and mediating variables were consistent in both models, involving two forms of work–family conflict, occupational burnout, and depression. Cognitive reappraisal was introduced as a moderating variable in the moderated mediation model.

## 3 Results

### 3.1 Common methods bias

This study collected data through self-report, which may lead to common method bias issues affecting the research results. Therefore, it was necessary to examine the presence of common method bias. Harman single-factor analysis was employed for detecting common method bias (Podsakoff et al., 2003). The results indicated seven factors with eigenvalues greater than 1, and the largest factor explained a variance of 30.87%, below the critical value of 40%. Hence, it can be inferred that this study did not have significant common method bias issues.

### 3.2 Collinearity test

Simultaneously, the process plug-in creates a mediating/moderating effect model using linear regression principles, resulting in a notable correlation among different variables in this process. Therefore, conducting a collinearity test holds great significance. The variance inflation factor (VIF) for all predictive variables (ranging from 1.080 to 1.505) was found to be less than 3. Additionally, the tolerance values (ranging from 0.657 to 0.926) were greater than 0.1. These results indicate that there is no significant issue of multicollinearity in the data, as reported by Dormann et al. (2013).

### 3.3 Descriptive analysis and correlation test of core variables

Descriptive statistics and correlation analysis revealed that work–family conflict was significantly positively related with family–work conflict, depression, and job burnout. The family–work conflict was positively related with depression and job burnout. Depression was positively correlated with job burnout. Additionally, cognitive reappraisal showed significant negative correlations with work–family conflict, family–work conflict, depression, and job burnout (Table 1).

**Table 1:** Mean, standard deviation and correlation coefficients of the variables.

Variable	M ± SD	1	2	3	4	5	6	7
Gender	1.77 ± 0.42	—						
Age	37.26 ± 9.40	−0.281***	—					
Work–family conflict	3.48 ± 0.91	−0.004	−0.011	—				
Family–work conflict	2.68 ± 0.91	−0.060**	−0.025	0.497***	—			
Depression	1.84 ± 0.62	0.041	−0.115***	0.431***	0.439***	—		
Job burnout	2.86 ± 0.46	0.025	−0.160***	0.475***	0.478***	0.611***	—	
Cognitive reappraisal	5.59 ± 1.05	−0.008	0.075***	−0.073**	−0.178***	−0.325***	−0.189***	—

**p < 0.01, ***p < 0.001.

### 3.4 Tests of mediating effects of depression

#### 3.4.1 Test of the mediate effect of depression on the relationship between work-to-family conflict and job burnout

Using model 4 from the SPSS PROCESS plugin, after controlling for gender, age, and family–work conflict, it was found that work–family conflict significantly predicted depression (*β* = 0.265, *SE* = 0.022, *p* < 0.001) and Job Burnout (*β* = 0.177, *SE* = 0.019, *p* < 0.001). Depression significantly predicted teacher job burnout (*β* = 0.439, *SE* = 0.018, *p* < 0.001). Further analysis using the bias-corrected Bootstrap method (*n* = 5,000) revealed a significant mediating effect of depression with a value of 0.116, *SE* = 0.012, 95% CI [0.093, 0.139]. Therefore, depression significantly mediated the relationship between work–family conflict and teacher job burnout (Table 2).

**Table 2:** Test of the mediate effect of depression.

		*β*	SE	*t*	95% CI	*R* ^2^	*F*
Equation 1: Depression	Work–family conflict	0.265	0.022	11.978***	[0.222, 0.308]	0.245	176.801***
	Gender	0.049	0.046	1.046	[−0.042, 0.139]		
	Age	−0.009	0.002	−4.140***	[−0.013, −0.005]		
	Family–work conflict	0.289	0.022	13.012***	[0.245, 0.332]		
Equation2: Job Burnout	Work–family conflict	0.177	0.019	9.226***	[0.139, 0.215]	0.467	381.811***
	Depression	0.439	0.018	24.399***	[0.404, 0.475]		
	Gender	−0.044	0.039	−1.119	[−0.120, 0.033]		
	Age	−0.009	0.002	−5.243***	[−0.013, −0.006]		
	Family–work conflict	0.208	0.019	10.718***	[0.170, 0.245]		

**p < 0.01, ***p < 0.001.

#### 3.4.2 Test of the mediate effect of depression on the relationship between family-to-work conflict and job burnout

**Figure 2:**
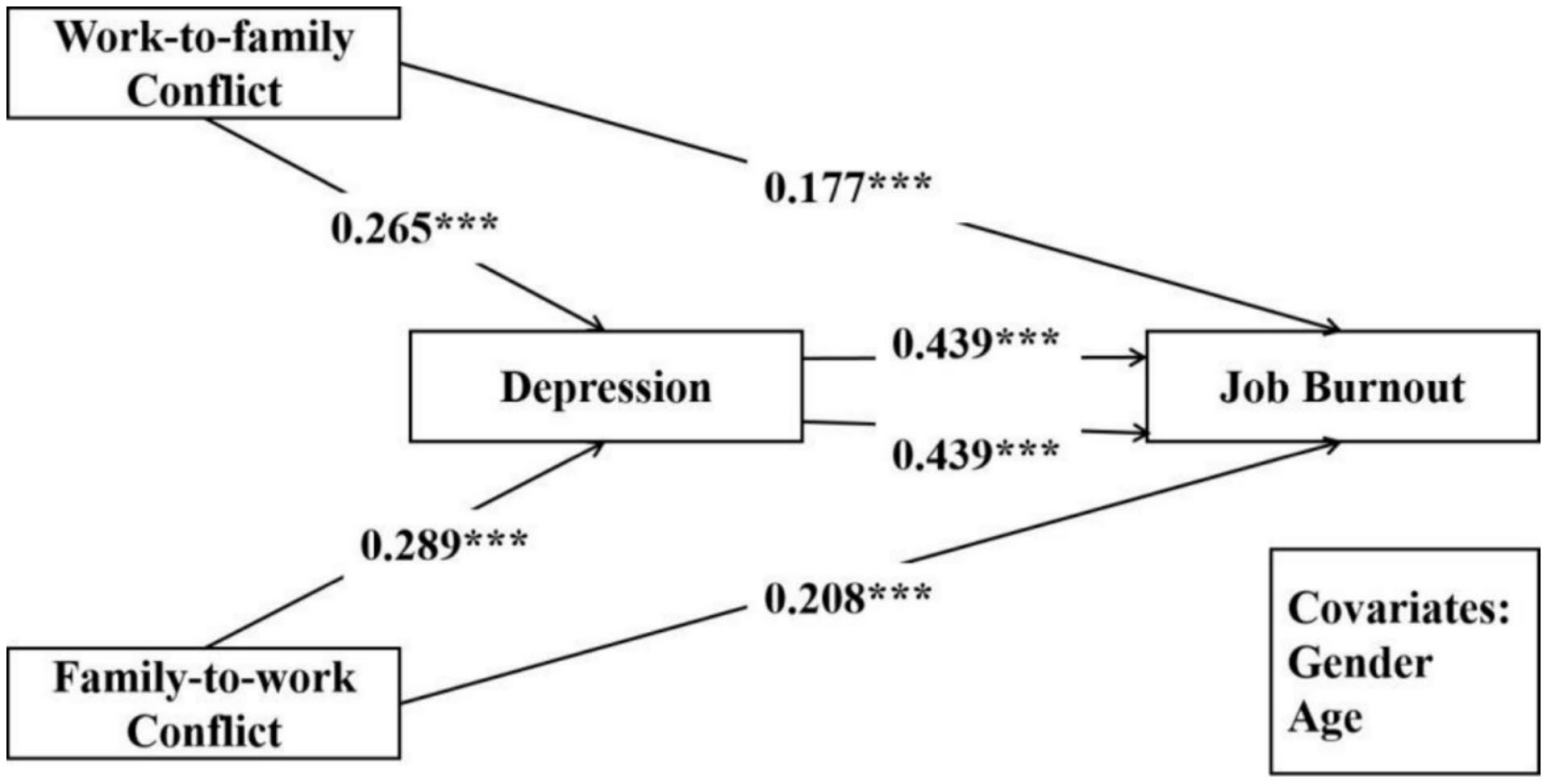
Mediating effects of depression.

After controlling for gender, age, and work–family conflict, it was observed that family–work conflict was significantly related to depression (*β* = 0.289, SE = 0.022, *p* < 0.001) and job burnout (*β* = 0.208, SE = 0.019, *p* < 0.001). Depression also significantly predicted teacher job burnout (*β* = 0.439, SE = 0.018, *p* < 0.001). The analysis using the bias-corrected Bootstrap method (*n* = 5,000) showed a significant mediating effect of depression with a value of 0.127, SE = 0.014, 95% CI [0.101, 0.155]. Thus, depression significantly mediated the relationship between family–work conflict and teacher job burnout. Details are shown in Figure 2 and Table 3.

**Table 3:** Test of the mediate effect of depression.

		*β*	SE	*t*	95% CI	*R* ^2^	*F*
Equation1: Depression	Family–work conflict	0.289	0.022	13.012***	[0.245, 0.332]	0.245	176.801***
	Gender	0.049	0.046	1.046	[−0.042, 0.332]		
	Age	−0.009	0.002	−4.140***	[−0.013, −0.005]		
	Work–family conflict	0.265	0.022	11.978***	[0.222, 0.308]		
Equation2: Job burnout	Family–work conflict	0.208	0.019	10.718	[0.170, 0.245]	0.467	381.811***
	Depression	0.439	0.018	24.399	[0.404, 0.475]		
	Gender	−0.044	0.039	−1.119	[−0.120, 0.033]		
	Age	−0.009	0.002	−5.243	[−0.013, −0.006]		
	Work–family conflict	0.177	0.019	9.226	[0.139, 0.215]		

**p < 0.01, ***p < 0.001.

### 3.5 Test of the moderate effect of cognitive reappraisal on the relationship between two different conflicts and job burnout

Using model 14 in the SPSS PROCESS plugin, a moderated mediation analysis was conducted. After controlling for gender, age, and family–work conflict, the results in Table 4 indicated a significant interaction effect where depression and cognitive reappraisal predict job burnout (*β* = 0.055, *SE* = 0.015, *p* < 0.001). This suggests that cognitive reappraisal moderates the latter part of the mediation pathway “Work–family Conflict → Depression → Job Burnout.”

**Table 4:** The moderate effect of cognitive reappraisal on the relationship between work-to- family conflict and job burnout.

		*β*	SE	*t*	95% CI	*R* ^2^	*F*
Equation 1: depression	Work–family conflict (X)	0.265	0.022	11.978***	[0.222, 0.308]	0.245	176.801***
	Gender	0.049	0.046	1.046	[−0.042, 0.139]		
	Age	−0.009	0.002	−4.140***	[−0.013, −0.005]		
	Family–work conflict	0.289	0.022	13.012***	[0.245, 0.332]		
Equation 2: Job burnout	Work–family conflict (X)	0.173	0.019	9.023***	[0.135, 0.210]	0.472	278.112***
	Depression (M)	0.455	0.019	24.652***	[0.419, 0.492]		
	Cognitive (W)	0.043	0.016	2.674**	[0.012, 0.075]		
	M × W	0.055	0.015	3.745***	[0.026, 0.084]		
	Gender	−0.044	0.039	−1.121	[−0.120, 0.033]		
	Age	−0.009	0.002	−5.212***	[−0.013, −0.006]		
	Family–work conflict	0.205	0.019	10.582***	[0.167, 0.243]		

**p < 0.01, ***p < 0.001.

Similarly, employing model 14 in the SPSS PROCESS plugin, after controlling for gender, age, and work–family conflict, the analysis shown in Table 5 revealed another significant interaction effect where depression and cognitive reappraisal predict job burnout (*β* = 0.005, SE = 0.015, *p* < 0.001). This indicates that cognitive reappraisal moderates the latter part of the mediation pathway “Family–work Conflict → Depression → Job Burnout.”

**Table 5:** The moderate effect of cognitive reappraisal on the relationship between family-to-work conflict and job burnout.

		*β*	SE	*t*	95% CI	*R* ^2^	*F*
Equation1: Depression	Family–work conflict (X)	0.289	0.022	13.012***	[0.245, 0.332]	0.245	176.801***
	Gender	0.049	0.046	1.046	[−0.042, 0.139]		
	Age	−0.009	0.002	−4.140***	[−0.013, −0.005]		
	Work–family conflict	0.265	0.022	11.978***	[0.222, 0.308]		
Equation2: Job burnout	Family–work conflict (X)	0.205	0.019	10.582***	[0.167, 0.243]	0.472	278.112***
	Depression (M)	0.455	0.019	24.652***	[0.419, 0.492]		
	Cognitive reappraisal (W)	0.043	0.016	2.674**	[0.012, 0.075]		
	M × W	0.055	0.015	3.745***	[0.026, 0.084]		
	Gender	−0.044	0.039	−1.121	[−0.120, 0.033]		
	Age	−0.009	0.002	−5.212***	[−0.013, −0.006]		
	Work–family conflict	0.173	0.019	9.023***	[0.135, 0.210]		

**p < 0.01, ***p < 0.001.

**Figure 3:**
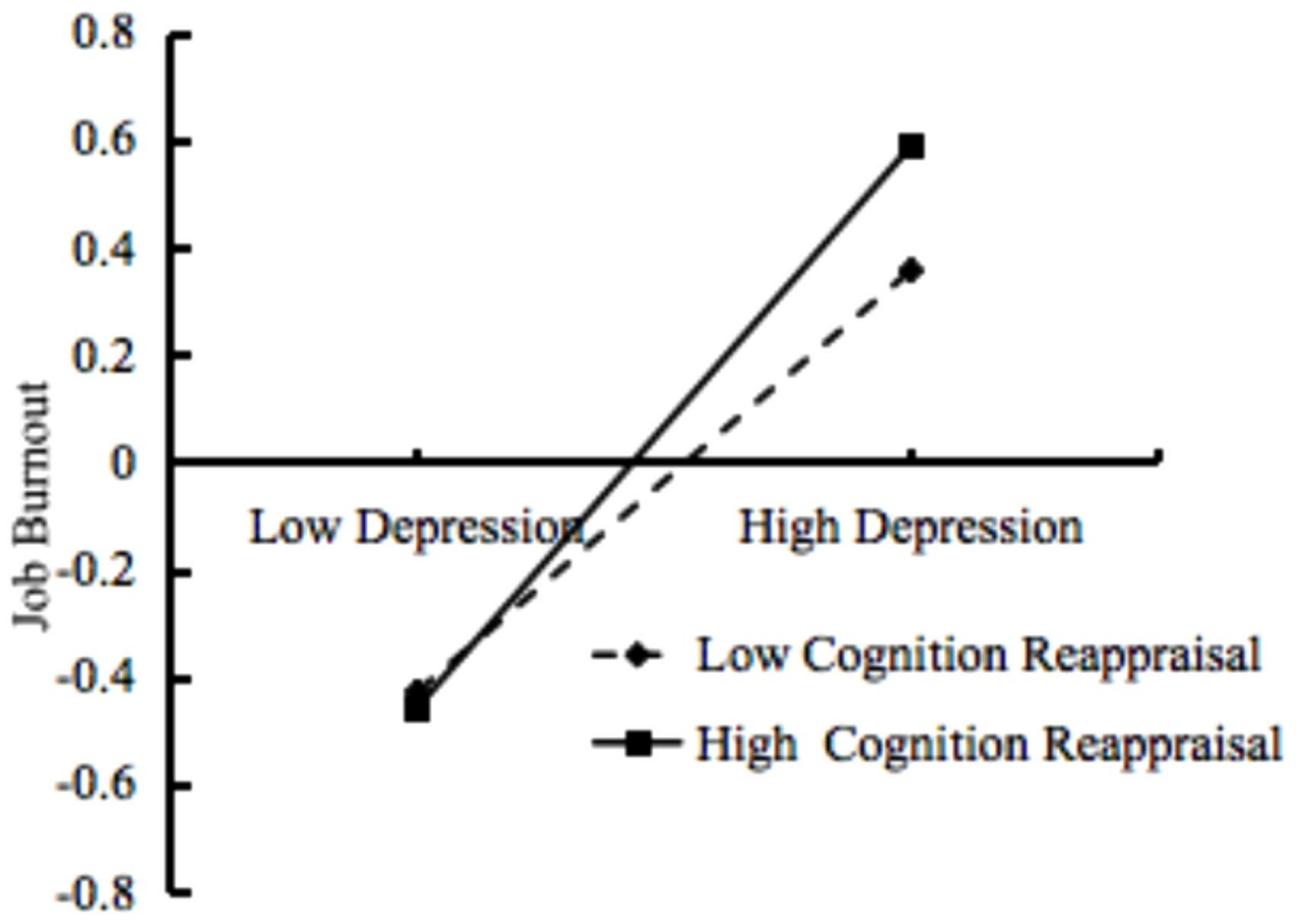
The moderating effect of cognitive reappraisal in the relationship between depression and job burnout.

To elucidate the substantial interactive effect of depression and cognitive reappraisal on job burnout, a simple slope test was further conducted. The effect value of depression on job burnout was calculated when cognitive reappraisal was one standard deviation above and below the mean (Mean ± SD). A simple effect graph was then plotted using the values obtained by taking the mean plus and minus one standard deviation for both depression and cognitive reappraisal based on the regression equation. Interestingly, the study results indicate that in two different forms of work-family models with job burnout as the moderated mediator, the relationship between depression and job burnout is moderated by cognitive reappraisal and the values are consistent. As shown in Figure 3, in the moderated mediation model of work–family conflict and teacher job burnout, the positive association between depression and job burnout is significantly moderated by cognitive reappraisal (*β* = 0.055, 95% CI: [0.026, 0.084], *p* < 0.001). Simultaneously, in the moderated mediation model of family–work conflict and teacher job burnout, the positive association between depression and job burnout is also significantly moderated by cognitive reappraisal (*β* = 0.055, 95% CI: [0.026, 0.084], *p* < 0.001). With an increase in cognitive reappraisal level, the relationship between depression and job burnout is enhanced (*β* = 0.398, *t* = 17.140, *p* < 0.001) VS (*β* = 0.530, *t* = 18.869, *p* < 0.001).

## 4 Discussion

Based on the conservation of resources theory, the affective events theory, and the stress-buffering model (Cohen and Wills, 1985; Grandey and Cropanzano, 1999; Weiss and Cropanzano, 1996), a moderated mediation model was established to explore the relationship between different types of work–family conflict among primary and secondary school teachers and job burnout, as well as the underlying mechanisms and whether there are differences between the two mechanisms. The results of the study indicate that both forms of work–family conflict are significantly positively related to job burnout among primary and secondary school teachers. Controlling for gender, age, and relative work–family conflict, both forms of work–family conflict can increase job burnout among primary and secondary school teachers by increasing levels of depression. Additionally, cognitive reappraisal significantly moderates the relationship between depression and job burnout.

Firstly, the research results support H1, indicating that both forms of work–family conflict are significantly positively related to job burnout among primary and secondary school teachers. This is consistent with previous research findings (Junca-Silva and Freire, 2022; Pu et al., 2017; Shang, 2022), reaffirming that as work–family conflict intensifies, the risk of job burnout for individuals also increases accordingly. Specifically, both WFC and FWC have negative impacts on teachers’ Job Burnout (Li et al., 2021). As emphasized by the conservation of resources theory (Grandey and Cropanzano, 1999), individuals have limited resources when coping with stress and challenges. When facing work–family conflict, individuals continuously need to allocate these limited resources to balance pressures from both work and family. If these consumed resources are not replenished in time, it can affect the individual’s resource allocation at work, ultimately leading to the emergence of Job Burnout (Hobfoll, 2002; Zhao et al., 2022). Therefore, paying attention to and alleviating work–family conflict among primary and secondary school teachers is of vital importance in reducing their job burnout.

Secondly, this study found that depression partially mediated the relationship between two different forms of work–family conflict and job burnout among primary and secondary school teachers, supporting H2. Prior research has indicated that depression is independently associated with both work–family conflict and job burnout (Peter et al., 2016; Wang and Peng, 2017; Zeng and Guo, 2024; Zhang et al., 2023). This study extends previous research by revealing the developmental path from work–family conflict to depression to job burnout among primary and secondary school teachers. These results suggest that experiencing two different forms of work–family conflict significantly impairs the psychological health of primary and secondary school teachers, increases their risk of depression, and thereby promotes the occurrence of job burnout. According to the affective events theory (Weiss and Cropanzano, 1996), work–family conflict as a negative work event triggers intense negative emotional experiences in individuals, such as depression (Zhou et al., 2018). Prolonged states of depression continuously deplete individuals’ limited psychological and emotional resources, leading to a decrease in their job satisfaction and enthusiasm, thereby triggering job burnout (Hatch et al., 2019; Zeng and Guo, 2024). Although a four-year longitudinal study found that family–work conflict is related to more symptoms of depression, while work–family conflict is unrelated to depression symptoms (Frone et al., 1997b), our study and some other studies indicate that the impact of the two different forms of work–family conflict on mental health is similar, both exacerbating depression symptoms (Hao et al., 2015; Zhou et al., 2020). This inconsistency reflects that the relative importance of work and family in individuals’ lives may dynamically change over time, cultures, and social contexts (Zhou et al., 2020).

Finally, this study revealed that cognitive reappraisal plays an important moderating role in the relationship between depression and job burnout, regardless of the influence in the WFC or FWC context, supporting H3. Specifically, when the level of depression is low, teachers with low cognitive reappraisal show higher levels of job burnout compared to those with high cognitive reappraisal; conversely, when depression levels are relatively high, the situation is reversed. This is inconsistent with the stress-buffering model (Cohen and Wills, 1985) and most previous studies (Feinberg et al., 2020; Shapero et al., 2019), but confirms researchers’ growing concern that Cognitive Reappraisal may be a double-edged sword, also having adverse effects (Feinberg et al., 2020; Troy et al., 2013). At lower levels of depression, cognitive reappraisal helps teachers maintain a positive attitude (Gross, 2014), effectively alleviating the negative impact of job burnout. However, at relatively higher levels of depression, overreliance or improper use of cognitive reappraisal may lead teachers to overlook the root of the problem, weaken the guiding and promoting role of negative emotions on appropriate social behavior (Forgas, 2013; Ketelaar and Tung, 2003), thereby reducing their motivation to take practical action to solve problems (Feinberg et al., 2020), exacerbating job burnout. Therefore, in coping with work–family conflict and depression, teachers need to carefully and flexibly choose emotional regulation strategies.

Although this study has theoretical and practical value in reducing job burnout among primary and secondary school teachers, we must acknowledge its limitations. Firstly, the study mainly relied on self-reported data collection methods, which may have certain methodological biases. To minimize this issue, we conducted statistical tests on methodological biases and found their impact to be insignificant. Future research could explore more diverse research methods, such as momentary ecological studies, to objectively investigate the relationship between work–family conflict and job burnout among primary and secondary school teachers. Secondly, due to the cross-sectional design of this study, we cannot draw strong causal conclusions and lack an in-depth understanding of the long-term dynamic relationships between different forms of work–family conflict, depression, and job burnout. Therefore, future research should employ longitudinal designs to examine causal and dynamic relationships between variables by tracking and collecting data at multiple time points.

Thirdly, the differences in teaching levels and the age stages of the students between middle school teachers and elementary school teachers may influence the research outcomes. Future studies could consider separately comparing these three distinct groups: junior high school teachers, senior high school teachers, and elementary school teachers, to further explore the differences in their models related to work–family conflict, depression, and job burnout. Such a nuanced analysis would provide a more comprehensive understanding of the mental health and influencing factors among teachers at different educational stages. Lastly, this study focused on Chinese primary and secondary school teachers as the sample, and its conclusions may be influenced by the specific educational environment and cultural background of China. To verify the generalizability of these findings, future research should further expand the sample to include teacher populations from more countries and regions, and compare the impact mechanisms of work–family conflict on job burnout in different cultural contexts.

## 5 Conclusion

This study was the first to examine the factors influencing job burnout in primary and secondary school teachers in China by integrating two different forms of work–family conflict, depression, and cognitive reappraisal. These findings had an important theoretical and practical implications for understanding and revealing the mechanisms of job burnout in primary and secondary school teachers. Firstly, this study found a positive predictive effect of work–family conflict on job burnout in primary and secondary school teachers, emphasizing that interventions aimed at reducing work–family conflict are conducive to promoting the psychological health and professional development of Chinese primary and secondary school teachers. Schools should create a positive work atmosphere for teachers, and strictly enforce work schedules to ensure teachers have enough rest time to maintain a good family and emotional life.

Besides, the mediating role of depression between work–family conflict and job burnout in primary and secondary school teachers suggests the need to establish a reliable psychological health screening mechanism for teachers, to regularly assess teachers’ psychological health and job burnout levels at different stages, in order to identify the presence of psychological problems and job burnout issues at an early stage. Furthermore, research findings on the regulatory role of cognitive reappraisal between depression and job burnout indicate that in work and life, primary and secondary school teachers should use cognitive reappraisal as an emotion regulation strategy to adjust the impact of depression on job burnout.

## Data Availability

The datasets generated during and/or analysed during the current study are available from the corresponding author on reasonable request.

## Footnotes

^1^www.wjx
